# Differential roles of prostaglandin E-type receptors in activation of hypoxia-inducible factor 1 by prostaglandin E_1_ in vascular-derived cells under non-hypoxic conditions

**DOI:** 10.7717/peerj.220

**Published:** 2013-11-28

**Authors:** Kengo Suzuki, Kenichiro Nishi, Satoshi Takabuchi, Shinichi Kai, Tomonori Matsuyama, Shin Kurosawa, Takehiko Adachi, Takayuki Maruyama, Kazuhiko Fukuda, Kiichi Hirota

**Affiliations:** 1 Department of Anesthesia, Kyoto University Hospital, Kyoto, Japan; 2 Department of Anesthesiology, Tohoku University Hospital, Sendai, Japan; 3 Department of Anesthesiology, Kansai Medical University, Hirakata, Osaka, Japan; 4 Department of Anesthesia, Tazuke Kofukai Medical Research Institute Kitano Hospital, Osaka, Japan; 5 Minase Research Institutes, Research Headquarters, Ono Pharmaceutical, Osaka, Japan

**Keywords:** Prostaglandin E1, Angiogenesis, VEGF, Hypoxia-inducible factor, EP receptor, Human endothelial cells, Human smooth muscle cells

## Abstract

Prostaglandin E_1_ (PGE_1_), known pharmaceutically as alprostadil, has vasodilatory properties and is used widely in various clinical settings. In addition to acute vasodilatory properties, PGE_1_ may exert beneficial effects by altering protein expression of vascular cells. PGE_1_ is reported to be a potent stimulator of angiogenesis via upregulation of VEGF expression, which is under the control of the transcription factor hypoxia-inducible factor 1 (HIF-1). However, the molecular mechanisms behind the phenomenon are largely unknown. In the present study, we investigated the mechanism by which PGE_1_ induces HIF-1 activation and VEGF gene expression in human aortic smooth muscle cells (HASMCs) and human umbilical vein endothelial cells (HUVECs), both vascular-derived cells. HUVECs and HASMCs were treated with PGE_1_ at clinically relevant concentrations under 20% O_2_ conditions and HIF-1 protein expression was investigated. Expression of HIF- 1α protein and the HIF-1-downstream genes were low under 20% O_2_ conditions and increased in response to PGE_1_ treatment in both HUVECs and HASMCs in a dose- and time-dependent manner under 20% O_2_ conditions as comparable to exposure to 1% O_2_ conditions. Studies using EP-receptor-specific agonists and antagonists revealed that EP1 and EP3 are critical to PGE_1_-induced HIF-1 activation. *In vitro* vascular permeability assays using HUVECs indicated that PGE_1_ increased vascular permeability in HUVECs. Thus, we demonstrate that PGE_1_ induces HIF- 1α protein expression and HIF-1 activation under non-hypoxic conditions and also provide evidence that the activity of multiple signal transduction pathways downstream of EP1 and EP3 receptors is required for HIF-1 activation.

## Introduction

Hypoxia-inducible factor 1 (HIF-1) is a transcription factor originally identified as a molecule responsible for hypoxia-induced expression of erythropoietin (Wang et al., 1995). More recently, the delineation of molecular mechanisms of angiogenesis has revealed a critical role for HIF-1 in the regulation of angiogenic growth factors. HIF-1 is identified as a critical factor in developmental, adaptive and pathological angiogenesis (Hirota & Semenza, 2006).

HIF-1 is a heterodimeric DNA-binding complex composed of two basic helix-loop-helix proteins of the PER-ARNT-SIM (PAS) family: HIF-1α and HIF-1β (Wang et al., 1995). The regulation of HIF-1 activity occurs at multiple levels in vivo. Hypoxia induces changes in the hydroxylation status of well-conserved prolyl and asparaginyl residues of HIF-1α, resulting in protein stabilization and transcriptional activation of HIF-1α (Hirota & Semenza, 2005; Schofield & Ratcliffe, 2005). Physiological stimuli other than hypoxia can also induce HIF-1 activation and transcription of hypoxia-inducible genes (Dery, Michaud & Richard, 2005; Nizet & Johnson, 2009; Pouyssegur & Mechta-Grigoriou, 2006). Signaling via receptor tyrosine kinases can induce HIF-1 expression by a mechanism independent of the presence of hypoxia. HER2/neu and IGF-1 receptor activation increases the rate of HIF-1α protein synthesis (Fukuda et al., 2002; Laughner et al., 2001).

Prostaglandins are a member of a group of lipid compounds that are derived enzymatically from fatty acids and have important functions in the animal body (Coleman, Smith & Narumiya, 1994). They are mediators and have a variety of strong physiological effects such as regulating the tone of smooth muscle tissue.

Among prostanoids, the E type prostaglandins, particularly PGE_2_ derived from arachidonic acid, are most widely produced in the body (Coleman, Smith & Narumiya, 1994). Molecular identification of the E type prostaglandins receptors was achieved by their cDNA cloning (Narumiya, Sugimoto & Ushikubi, 1999; Sugimoto & Narumiya, 2007), which revealed that the receptors are G-protein-coupled receptors (GPCRs).

Prostaglandin E_1_ (PGE_1_), known pharmaceutically as alprostadil, has vasodilatory properties. In addition to acute vasodilatory properties, PGE_1_ may exert additional effects by altering protein expression of endothelial cell and other types of cells. For example, PGE_1_ is reported to be effective for treating patients with severe hepatitis and primary graft non-function after liver transplantation (Andrade Jr, Andrade & Santos, 2009). Moreover, it is reported that PGE_1_ is a potent stimulator of angiogenesis via upregulation of VEGF expression (Haider et al., 2005; Inoue et al., 2013; Mehrabi et al., 2003). However, the molecular mechanisms behind the phenomena are as yet largely unknown. Earlier studies (Fukuda, Kelly & Semenza, 2003; Han, Michalopoulos & Wu, 2006; Ji et al., 2010) revealed the mechanism by which PGE_2_ exposure induces vascular endothelial growth factor (VEGF) gene expression in HCT116 human colon carcinoma cells, EP1 receptor-overexpressed HE293 cells and Hep2G cells. PGE_2_ induces the expression of HIF-1α protein and EP1 receptor activation appears to be necessary and sufficient to induce HIF-1α expression in HCT116 cells adopting rather higher concentration of the EP1 agonist 17-pt-PGE_2_ and the antagonist 50 µM SC-51322 and 1 µM sulprostone. In contrast, it is reported that PGE_1_ causes an upregulation of eNOS and VEGF protein and mRNA expression and decreases HIF-1α in human umbilical vein endothelial cells (HUVECs) without demonstration of molecular mechanism behind the observation (Haider et al., 2005). Moreover, there is a report describing that alprostadil suppresses angiogenesis (Cattaneo et al., 2003). Thus, involvement of the type E prostaglandin especially PGE_1_ in VEGF secretion and the subsequent angiogenesis and the molecular mechanisms behind the phonemenon is largely unknown. The evidence prompted us to investigate the effect of PGE_1_ on HIF-1 activation, VEGF secretion and the initial steps of angiogenesis adopting smooth muscle cells and endothelial cells.

In the present study, we investigated the mechanism by which PGE_1_ induces VEGF gene expression in human aortic smooth muscle cells (HASMCs) and HUVECs and demonstrated that PGE_1_ induces HIF-1 activation by the increasing expression of HIF-1α protein and its downstream genes including VEGF in an EP1 and EP3 prostaglandin receptors-dependent manner. In addition, we aimed to elucidate the consequence of PGE_1_-induced HIF-1 dependent VEGF secretion on the initial step of angiogenesis using human endothelial cells.

## Materials and Methods

### Cell culture and reagents

Human aortic smooth muscle cells (HASMCs) and human umbilical vein endothelial cells (HUVECs) were purchased from Kurabo (Osaka, Japan) (Oda et al., 2009). Human neuroblastoma cell line SH-SY5Y cells obtained from ATCC (Manassas, VA), were maintained in RPMI 1640 medium containing 10% fetal bovine serum (FBS) (Daijo et al., 2011; Takabuchi et al., 2004). HEK293 cells were maintained in Dulbecco’s Modified Eagle medium (DMEM) containing 10% fetal bovine serum (FBS) (Hirota et al., 2004). PGE_1_ was obtained from Sigma-Aldrich (St. Louis, MO). Lipo-PGE_1_ was from Mitsubishi Tanabe Pharma (Osaka, Japan). PGE_1_-Alfadex (7{-(1 R, 2 R , 3R )-3-Hydroxy-2[-(1 E, 3 S)-3-hydroxyoct-1-en-1-yl]-5-oxocyclopentyl} heptanoic acid-α-Cyclodextrin) was from Ono Pharmaceutical Co. (Osaka, Japan). Cycloheximide (CHX), desferrioxamine (DFX), LY294002, PD98059, and GF109203X were obtained from Calbiochem (San Diego, CA). YC-1 was obtained from Cayman Chemical Company (Ann Arbor, MI).

Anti-HIF-1α antibody was purchased from BD Biosciences (San Jose, CA) and anti-HIF-1β antibody was purchased from Novus Biologicals (Littleton, CO). Anti-β-actin antibody was purchased from Sigma-Aldrich.

### EP receptor-specific agonists and antagonists

The specific agonists, ONO-DI-004 for EP1, ONO-AE1-259-01 for EP2, ONO-AE-248 for EP3, ONO-AE1-329 for EP4 and the specific antagonists, ONO-8713 for EP1, ONO-AE3-240 for EP3, and ONO-AE2-227 for EP4 were kindly provided by Ono Pharmaceutical Co. (Suzawa et al., 2000).

### Hypoxic treatment

Cells were maintained in a multi-gas incubator (APMW-36, Astec, Japan) and exposed to hypoxia (1% O_2_, 5% CO_2_, and 94% N_2_ or 5% O_2_, 5% CO_2_, and 90% N_2_) at 37°C (Tanaka et al., 2010; Tanaka et al., 2011).

### Immunoblot assays

Whole cell lysates were prepared using ice-cold lysis buffer following a protocol described previously (Kasuno et al., 2004; Oda et al., 2006; Tanaka et al., 2010). Aliquots of the cell lysates were fractionated by 7.5% SDS-PAGE and subjected to immunoblot assay using mouse monoclonal antibody against HIF-1α (BD Biosciences, San Jose, CA) or HIF-1β (H1b234; Novus Biologicals, Littleton, CO) at 1:1000 dilution and HRP-conjugated mouse monoclonal antibodies against mouse IgG (Amersham Bioscience, Piscataway, NJ, 1:1000 dilution). Anti-β-actin antibody (Sigma, St. Louis, MO) was used at 1:5000 dilution. Chemiluminescent signals were developed using ECL reagent (Amersham Biosciences). The intensity of each band was quantified using Image J software (Oda et al., 2006).

### Metabolic labeling assay

The metabolic-labeling protocol is described elsewhere (Kasuno et al., 2004). Briefly, HASMCs were plated in a 10-cm dish and 24 h later were serum-starved for a further 12 h. The cells were pretreated with 10 µM PGE_1_ or 100 µM DFX for 30 min in methionine-free Dulbecco’s modified Eagle’s medium. [35S] Met-Cys was added to a final concentration of 0.3 mCi/ml, and the cells were pulse-labeled for 45 min and then harvested. Whole cell extracts were prepared and 1 mg of extract was precleared with 60 µl of protein A-Sepharose for 1 h. Anti-HIF-1 antibody (20 µl) was added to the supernatant and the mixture was rotated overnight at 4°C. Protein A-Sepharose (40 µl) was added and the mixture rotated for 2 h at 4°C, pelleted, and washed five times with 1 ml of radioimmune precipitation buffer. The samples were analyzed by SDS-polyacrylamide gel electrophoresis. The gel was dried and exposed to X-ray film.

### Quantitative real-time reverse transcriptase (RT)-PCR analysis (*q*RT-PCR)

RNA was purified using RNeasy™ (Qiagen, Valencia, CA) and treated with DNase. First-strand synthesis and real-time PCR were performed using the QuantiTect SYBR Green PCR Kit (Qiagen) following the manufacturer’s protocol. Amplification and detection were performed using the Applied Biosystems 7300 Real-time PCR System (Applied Biosystems, Foster City, CA) (Oda et al., 2008). PCR primers were purchased from Qiagen (Oda et al., 2009; Tanaka et al., 2010). The change in expression of each target mRNA relative to 18S rRNA was calculated.

### VEGF ELISA

Secreted VEGF protein levels in culture supernatant were determined with an ELISA kit (Quantikine Human VEGF ELISA kit; R & D Systems) according to the manufacturer’s instruction. Values of optical density were measured spectrophotometrically (Microplate Reader 680 XR; Bio-Rad) at 450 nm (correction wavelength set at 570 nm). The standard curve was made with Prism™ software. In each experiment, all samples and standards were measured in duplicate. The amount of VEGF released in the supernatant was expressed in pg/ml (Tanaka et al., 2011).

### *In vitro* vascular permeability assay

The assay kit was purchased from Milliore (Billerica, MA). HUVEC cells were seeded at 200,000 cells per insert and cultured for 72 h. HUVEC monolayer permeability was tested after a 24 h starvation period. Test inserts were then treated for 18 h with 1 µM PGE_1_ in basal medium. Monolayers were also treated with basal medium and growth medium only. Inserts were also tested without the cell monolayer. The fluorescence of the plate well solution was determined using a standard fluorescent plate reader.

### Transepithelial electrical resistance measurements

Measurement of transepithelial electrical resistance in HUVEC monolayer was performing using a Millicell electric resistance system (ERS) ohmmeter (Millipore Corporation, Bedford, MA) as a barrier function assay (Kimura et al., 2008). This device can measure electric resistance of epithelial cells in culture using a separate pair of Ag-AgCl electrolodes and a resistance meter. Fluid resistance was subtracted and net resistance was calculated as ohm-square centimeter. Results are presented as the mean ± S.D. of three independent wells.

### Statistical analysis

Data are presented as means ± S.D. Data were analyzed using one-way analysis of variance followed by Dunnett’s multiple comparison test using Prism version 4c. *P* < 0.05 was considered statistically significant (Tanaka et al., 2010; Tanaka et al., 2011).

## Results

### PGE_1_ induces HIF-1α protein accumulation in vascular-derived cells

**Figure 1:**
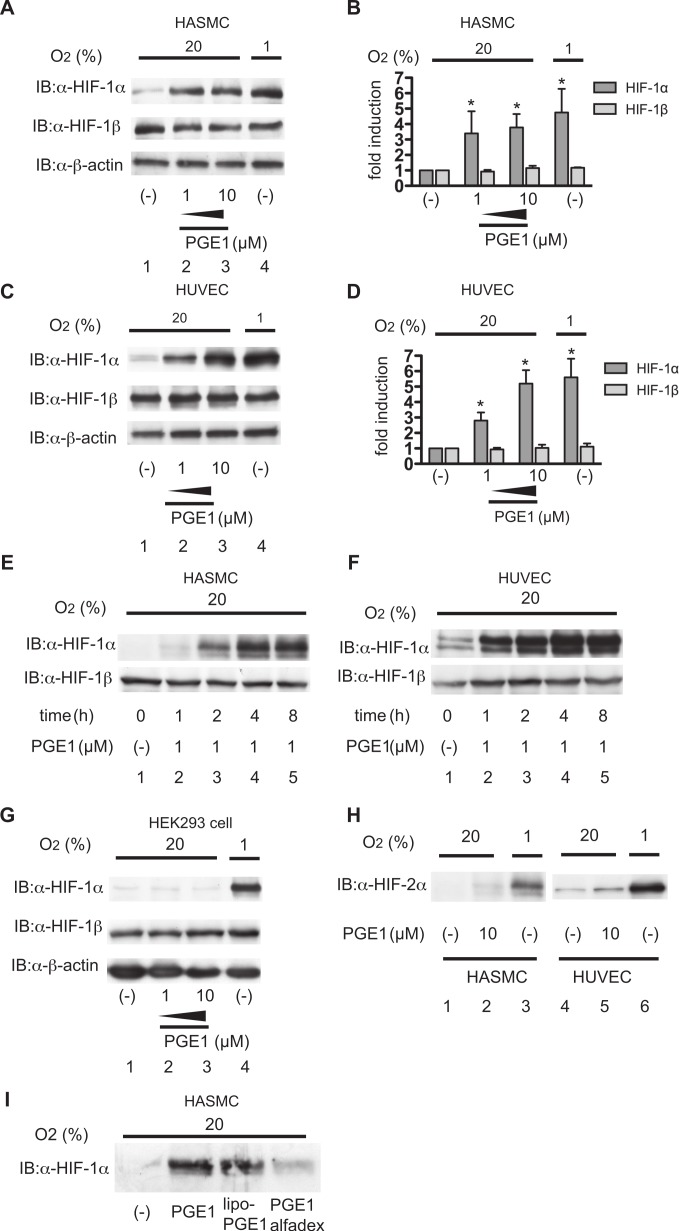
PGE_1_ induces HIF-1α protein accumulation in vascular-derived cells. Human aortic smooth muscle cells (HASMCs) (A and B), human umbilical vein endothelial cells (HUVECs) (C and D), and HEK293 cells (G) were exposed to 1 or 10 µM PGE_1_ under 20% O_2_ or 1% O_2_ conditions for 4 h. After treatment, cells were harvested and whole-cell lysates were subjected to immunoblot assay for HIF-1α, HIF-1β, and β-actin protein expression. Experiments were repeated thrice (A, C and G). Representative immunoblots are shown (A and C). Band intensities were analyzed densitometrically (B and D). Fold induction relative to lane 1 was plotted as mean ± S.D. ^∗^*P* < 0.05 compared with the control. HASMCs (E) and HUVECs (F) were exposed to 1 µM PGE_1_ for the indicated times under 20% O_2_ and were harvested for immunoblot assay for HIF-1α protein. Experiments were repeated twice. Representative immunoblots are shown. (H) HASMCs and HUVECs were exposed to 10 µM PGE_1_ for 4 h under 20% O_2_ and were harvested for immunoblot assay for HIF-2α protein. Experiments were repeated twice. Representative immunoblots are shown. I. HASMCs were exposed to 1 µM PGE_1_, lipo-PGE_1_ and PGE_1_-alfadex under 20% O_2_ conditions for 4 h. After treatment, cells were harvested and whole-cell lysates were subjected to immunoblot assay for HIF-1α.

With the aim of evaluating the effect of PGE_1_ (alprostadil) on HIF-1 activity, HASMCs and HUVECs were treated with or without 1 or 10 µM PGE_1_ under 20% O_2_ conditions for 4 h and HIF-1 protein expression was investigated (Figs. 1A–1D). HIF-1α protein expression was low under 20% O_2_ conditions (lane 1) and increased in response to PGE_1_ treatment in both HASMCs and HUVECs (lanes 2 and 3) as well as under 1% O_2_ conditions (lane 4) (Figs. 1A and 1C). HIF-1β protein expression was not affected by PGE_1_ treatment in either HASMCs or HUVECs. β-actin expression remained constant during treatment. Densitometric analysis showed that both 1 µM and 10 µM PGE_1_ statistically significantly induced HIF-1α protein expression as early as 4 h (Figs. 1B and 1D). With the aim of constructing a time course of the effect of PGE_1_ on HIF-1α protein accumulation, cells were exposed to 1 µM PGE_1_ for 2, 4, and 8 h. The time-course study demonstrated that 1 µM PGE_1_ induced HIF-1α protein expression as early as 1 h with peak activity at 4 h (lanes 4) in both HASMCs (Fig. 1E) and HUVECs (Fig. 1F). No toxic effect was detected as judged from an MTT assay (data not shown). PGE_1_ did not induce HIF-1α protein accumulation in HEK293 cells (Fig. 1G). In contrast, 1 µM PGE_1_ did not affect HIF-2α protein expression under 20% O_2_ conditions in either HASMCs or HUVECs at 4 h (Fig. 1H). There are several preparations of PGE_1_ available in clinical settings. We tested the two PGE_1_ analogues; lipo-PGE_1_ and PGE_1_-Alfadex (Fig. 1I). Both lipo-PGE_1_ and PGE_1_-Alfadex induced HIF-1α protein accumulation in HASMCs under normoxic conditions.

### PGE_1_ induces HIF-1 activation and expression of its downstream genes

**Figure 2:**
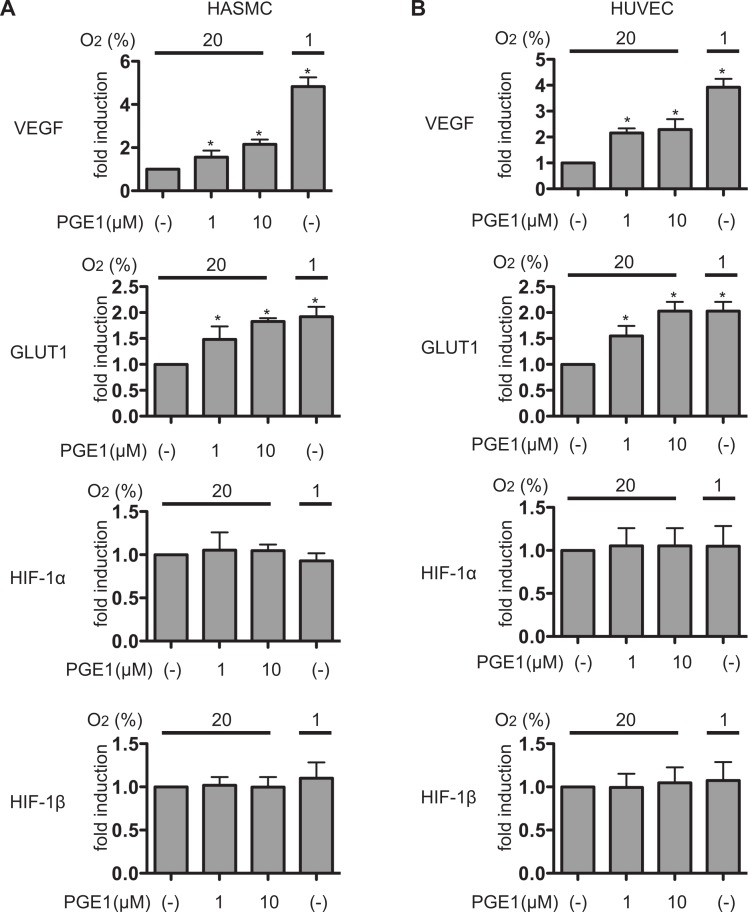
PGE_1_ induces HIF-1 activation and expression of its downstream genes. Human aortic smooth muscle cells (HASMCs) (A) and human umbilical vein endothelial cells (HUVECs) (B) were exposed to 1 µM or 10 µM PGE_1_ for 4 h under 20% or 1% O_2_ and harvested for semi-quantitative RT-PCR for vascular endothelial growth factor (VEGF), glucose transporter 1 (GLUT1), HIF-1α and HIF-1β. Experiments were repeated at least three times in triplicate. Fold induction relative to that under 20% O_2_ without PGE_1_ treatment was plotted. ^∗^*P* < 0.05 compared with the control (20% and no treatment).

**Figure 3:**
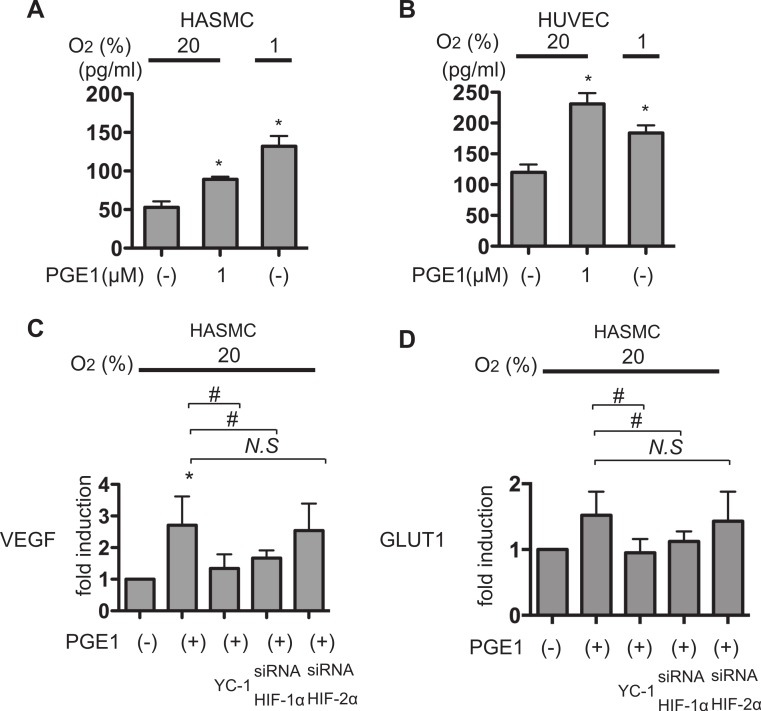
PGE_1_ induces VEGF secreation in an HIF-1α-dependent manner. HUVECs (A) and HASMCs (B) were exposed to 1 µM PGE_1_ for 4 h and supernatants of the culture media were subjected to ELISA for VEGF protein. The concentrations were indicated in pg/ml. ^∗^*P* < 0.05 compared with the control (20% and no treatment). HASMCs were exposed to 1 µM PGE_1_ with 10 µM YC-1 for 4 h under 20% or 1% O_2_ and harvested for semi-quantitative RT-PCR for VEGF (C) and GLUT1 (D). Fold induction relative to that under 20% O_2_ without PGE_1_ treatment was plotted. ^∗^*P* < 0.05 compared with the control (20% and no treatment), ^#^*P* < 0.05 for comparisons between the indicated groups.

We next investigated the effect of PGE_1_ on HIF-1-mediated gene expression. The mRNA expression of genes was assayed by qRT-PCR. PGE_1_ treatment induced VEGF and GLUT1 mRNA expression in a PGE1-dose dependent manner under 20% O_2_ conditions in both HASMCs (Fig. 2A) or HUVECs (Fig. 2B) at 4 h. The fold induction of VEGF mRNA by PGE1 was less than that of hypoxic exposure (1% O_2_ conditions). In contrast, the induction of GLUT1 mRNA was comparable to that of hypoxic exposure. This is consistent with the results of HIF-1α protein expression (Figs. 1A and 1C). The expression of either HIF-1α or HIF-1β mRNA was not affected by PGE_1_. Moreover, PGE_1_ induced VEGF protein secretion in both HASMCs (Fig. 3A) or HUVECs (Fig. 3B). The induction of VEGF mRNA in HASMCs was inhibited by the inhibitor of HIF-1 activation YC-1 or downregulation of HIF-1α expression by siRNA, indicating that the effect of PGE_1_ is dependent on HIF-1 activation (Fig. 3C). Glut1 mRNA expression was also affected by YC-1 and downregulation of HIF-1α expression by siRNA similarly to VEGF mRNA (Fig. 3D).

### Differential involvement of EP receptors in PGE_1_-induced HIF-1 activation

**Figure 4:**
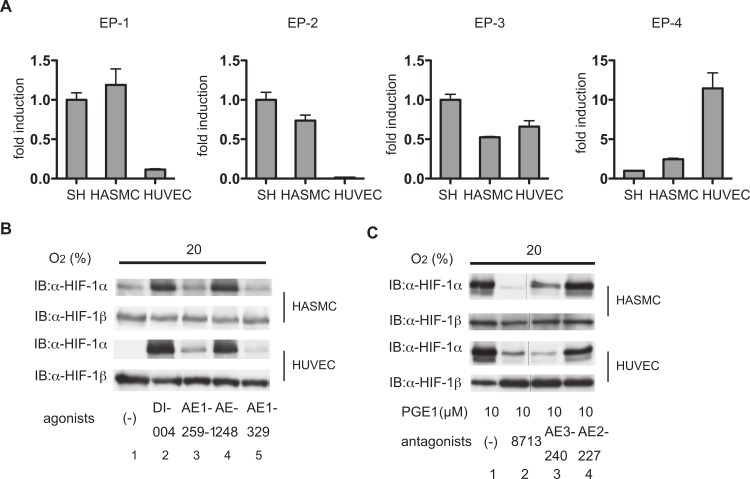
Differential involvement of EP receptors in PGE_1_-induced HIF-1α protein accumulation. (A) Expression of EP1, EP2, EP3, and EP4 receptors in human aortic smooth muscle cells (HASMCs), human umbilical vein endothelial cells (HUVECs), and cells of the neuroblastoma cell line SH-SY5Y. HASMCs, HUVECs, and SH-SY5Y cells were cultured under 20% O_2_ and harvested for semi-quantitative RT-PCR for EP1-4 receptors. Experiments were repeated at least three times in triplicate. Fold induction relative to expression in SH-SY5Y cells was plotted as mean ± S.D. HASMCs and HUVECs were exposed to 1 µM of EP-receptor-specific agonists (ONO-DI-004 for EP1, ONO-AE1-259-01 for EP2, ONO-AE-248 for EP3, and ONO-AE1-329 for EP4) for 4 h (B). HASMCs and HUVECs were exposed to 1 µM PGE_1_ with or without 1 µM EP-receptor-specific antagonists (ONO-8713 against EP1, ONO-AE3-240 against EP3, and ONO-AE2-227 against EP4) for 4 h (C). The cells were harvested and the whole-cell lysates were subjected to immunoblot assay for HIF-1α and β-actin protein expression. Experiments were repeated twice. Representative immunoblots are shown.

Four types of EP receptors have been identified for PGE_1_ (Sugimoto & Narumiya, 2007). We sought to identify those responsible for PGE_1_-induced HIF-1 activation. Expression of EP receptors in HUVECs and HSMACs was examined by qRT-PCR. It is reported that SH-SY5Y cells express EP1, EP2, EP3, and EP4 receptors (Ji et al., 2010). As shown in Fig. 4A, HASMCs express all types of EP receptor. HUVECs express the EP1, EP3, and EP4 receptors. The EP2 receptor was not detected in HUVECs.

**Figure 5:**
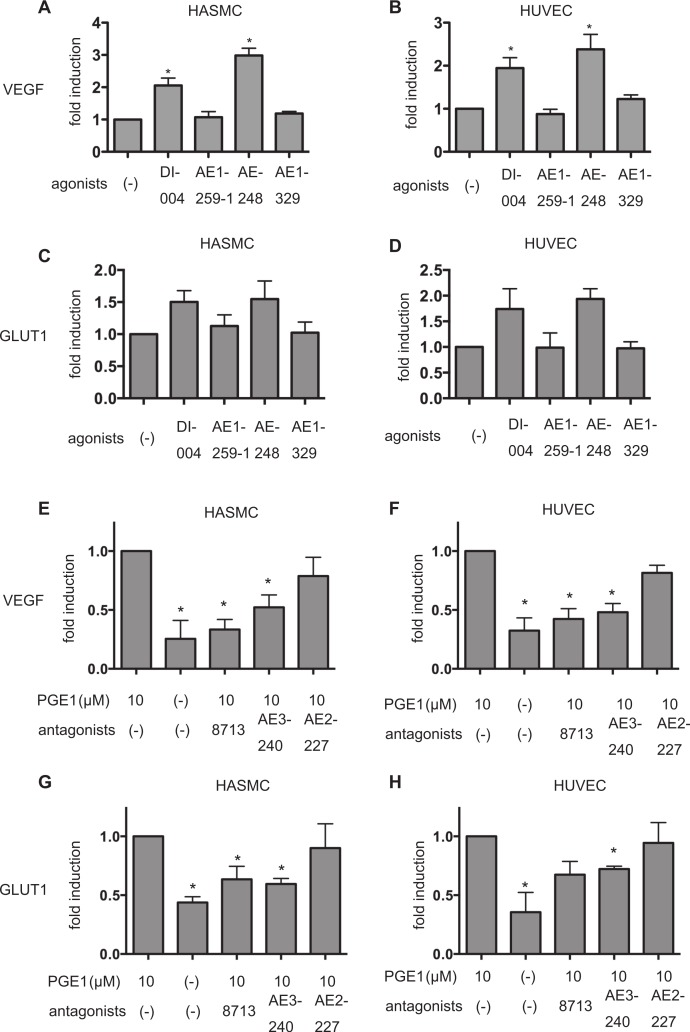
Differential involvement of EP receptors in PGE_1_-induced HIF-1 activation. HASMCs and HUVECs were exposed to 1 µM of EP-receptor-specific agonists for 4 h and harvested for semi-quantitative RT-PCR for VEGF (A) and GLUT1 (B). Fold induction relative to that under 20% O_2_ without PGE_1_ treatment was plotted. ^∗^*P* < 0.05 compared with the control (no treatment). HASMCs and HUVECs were exposed to 1 µM of EP-receptor-specific antagonists with 10 µM PGE_1_ for 4 h and harvested for semi-quantitative RT-PCR for VEGF (C) and GLUT1(D). Fold induction relative to that under 20% O_2_ without PGE_1_ treatment was plotted. ^∗^*P* < 0.05 compared with the control (no antagonist with PGE_1_).

Among EP receptor-subtype-specific ligands, the EP1-specific ligand ONO-DI-004 (Fig. 4B, lanes 2) and the EP3-specific ligand ONO-AE-248 (Fig. 4B; lanes 4) induced HIF-1α protein accumulation in both HASMCs and HUVECs. In contrast, neither the EP2-specific ligand ONO-AE1-259-01 (Fig. 4B; lanes 3) nor the EP4-specific ligand ONO-AE1-329 (Fig. 4B; lanes 5) induced HIF-1α protein expression at 1 µM concentration. Consistent with these observations, the EP1-specific antagonist ONO-8713 or the EP3-specific antagonist ONO-AE3-240 inhibited PGE_1_-induced HIF-1α protein accumulation in both HASMCs and HUVECs (Fig. 4C). HIF-1β expression was not affected by any agonists of the EP receptors (Fig. 4B). Moreover, *q*RT-PCR analysis showed that ONO-DI-004 and ONO-AE-248 induced expression of VEGF mRNA in both HASMCs (Fig. 5A) and HUVECs (Fig. 5B) at 4 h. *q*RT-PCR analysis showed that ONO-8713 and ONO-AE3-240 did suppress but the EP4-specific antagonist ONO-AE2-227 did not suppress VEGF or GLUT1 mRNA induction by PGE_1_ expression statistically significantly in both HASMCs (Fig. 5C) or HUVECs (Fig. 5D).

### Effect of PGE_1_ on stability and synthesis of HIF-1α

**Figure 6:**
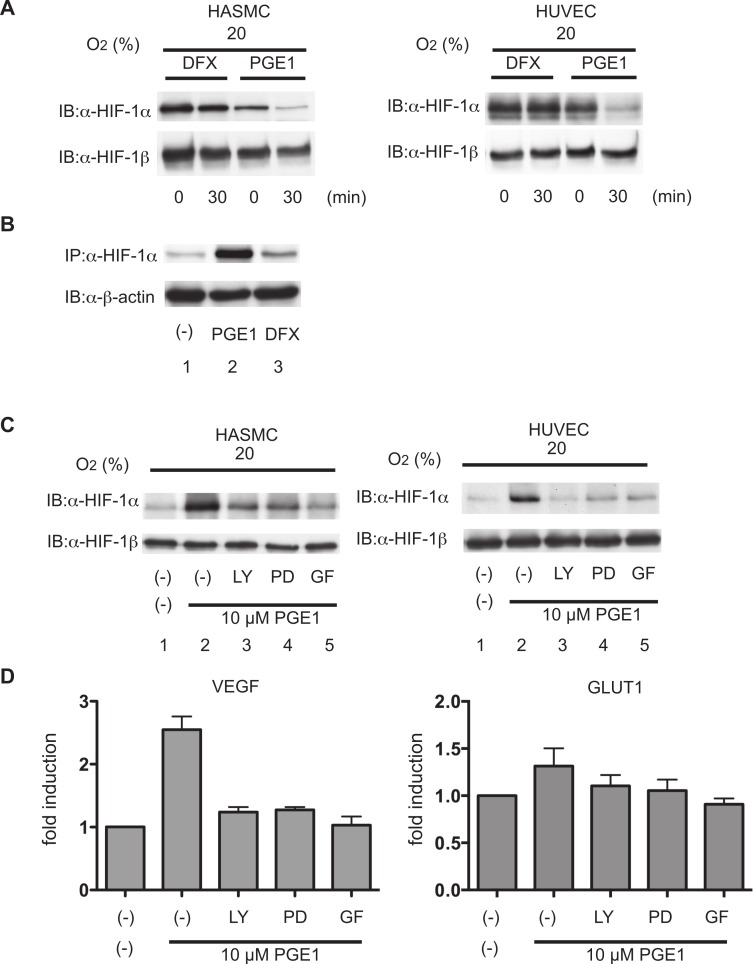
Effect of PGE_1_ on stability and synthesis of HIF-1α. (A) Human aortic smooth muscle cells (HASMCs) and human umbilical vein endothelial cells (HUVECs) were exposed to 1 µM PGE_1_ or 100 µM DFX or incubated for 4 h, and CHX was added to a final concentration of 100 µM. The cells were incubated for 0 to 30 min, and whole-cell lysates were subjected to immunoblot assay using anti-HIF-1α or -β antibodies. (B) Serum-starved HASMCs were pretreated with no drug and 1 µM PGE_1_ for 30 min in Met-free medium. [35S]Met-Cys was added, and the cells were incubated for 60 min prior to preparation of cell lysates. Aliquots of 1 mg of the lysates were subjected to immunoprecipitation with anti-HIF-1α antibody, separated by SDS-PAGE and exposed. Aliquots of 50 µg of the same lysate were separately subjected to immunoblotting analysis with anti-β-actin antibody. (C) HASMCs and HUVECs were exposed to vehicle or 1 µM PGE_1_ for 4 h in the presence of 10 µM LY294002 (LY), 50 µM PD98059 (PD), or 5 µM GF109203X (GF). The cells were harvested and the whole-cell lysates were subjected to immunoblot assay for HIF-1α and β-actin protein expression. Experiments were repeated at least twice. Representative immunoblots are shown. (D) HASMCs were exposed to vehicle or 1 µM PGE_1_ for 12 h in the presence of 10 µM LY294002 (LY), 50 µM PD98059 (PD), or 5 µM GF109203X (GF). Cells were harvested and subjected to semi-quantitative RT-PCR for VEGF and GLUT1. Experiments were repeated three times. Fold induction relative to that under 20% O_2_ without PGE_1_ treatment was plotted. ^∗^*P* < 0.05 compared with the control (20%, PGE1 treatment without any kinase inhibitors).

The balance between synthesis and degradation determines HIF-1α protein levels. With the aim of exploring the mechanism for HIF-1α protein induction by PGE_1_, HIF-1α protein half-life in the cells was examined. HASMCs and HUVECs were treated with PGE_1_ or DFX for 4 h to induce HIF-1α protein accumulation. Then, CHX was added to block ongoing protein synthesis. In the presence of CHX, HIF-1α protein expression was not detected at 30 min in PGE_1_-treated cells but persisted in DFX-treated cells in both HASMCs and HUVECs (Fig. 6A). These results strongly suggested that HIF-1α expression in PGE_1_-treated cells is dependent mainly upon ongoing protein synthesis.

With the aim of determining the rate of HIF-1α synthesis, serum-starved HUVECs were pretreated with PGE_1_ or DFX for 30 min and then pulse-labeled with [35S] Met-Cys for 40 min, followed by immunoprecipitation of HIF-1α (Fig. 6B). In contrast to control serum-starved cells, 35S-labeled HIF-1α clearly increased in PGE_1_-treated cells (lane 2), whereas the amount of labeled HIF-1α protein did not increase in cells treated with DFX (lane 3), relative to the control. Thus, both CHX addition and metabolic labeling experiments provide evidence for increased synthesis of HIF-1α in response to PGE_1_ treatment. Immunoblotting analysis against the same lysates with anti-β-actin antibody was indicated as loading control of the lysates (Fig. 6B).

To examine the signaling pathways leading to HIF-1α protein induction in HUVECs and HASMCs, we focused on various kinases, including PI3K, mitogen-activated protein kinase (MAPK), protein kinase C (PKC) and mammalian target of rapamycin (mTOR) (Fukuda, Kelly & Semenza, 2003).

HIF-1 activity induced by the stimulation of receptor tyrosine kinases or G protein-coupled receptors has been reported to require MAPK and PI3K signaling (Fukuda, Kelly & Semenza, 2003; Hirota et al., 2004). Cells were pretreated with LY294002, PD98059 or GF109203X, which are selective inhibitors of PI3K, MAPK and PKC, respectively, for 30 min, and then treated with 10 µM PGE_1_ for 4 h and harvested for immunoblot assay in both HASMCs and HUVECs (Fig. 6C). All the inhibitors inhibited the induction of HIF-1α protein expression by PGE_1_. HIF-1β protein expression was not induced nor affected by any of the kinase inhibitors. Consistent with kinase inhibitor-mediated inhibition of HIF-1α protein expression, LY294002, PD98059, or GF109203X also suppressed PGE_1_-induced VEGF and GLUT1 mRNA expression (Fig. 6D) in HASMCs.

### PGE_1_ increases permeability of vascular barrier reconstituted with HUVECs

**Figure 7:**
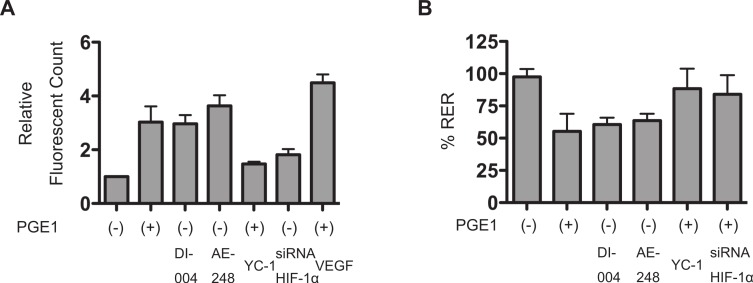
PGE_1_ increases permeability of vascular barrier reconstituted with HUVECs. HUVECs were seeded, allowed to form a monolayer for 72 h. (A) Cells were treated with 10 µM PGE_1_ for a further 18 h, after which the extent of permeability was determined as described in Materials and Methods. Fold induction relative to the control (cells without PGE_1_ treatment) was plotted as mean ± S.D. (*n* = 4). ^∗^*P* < 0.05 compared with the control. (B) Cells were treated for a further 18 h under 20% O_2_ conditions with conditioned media derived from HUVECs treated with 1 µM PGE_1_ with or without the treatment with YC-1 or siRNA against HIF-1α, ONO-DI-004, or ONO-AE-248 treatment for 24 h. Then, transepithelial electrical resistance was measured. Percent suppression of resistance relative to the control (cells without PGE_1_ treatment) was plotted as mean ± S.D. (*n* = 4). ^∗^*p* < 0.05 vs. control. RER, relative electrical resistance.

With the aim of determining whether PGE_1_ altered vascular barrier function, in vitro vascular permeability assay using HUVECs were performed (Fig. 5). HUVECs were seeded, allowed to form a monolayer for 72 h, and treated with or without 1 µM PGE_1_, the EP1-specific ligand ONO-DI-004 or the EP3-specific ligand ONO-AE-248 for a further 18 h under 20% O_2_ conditions and the extent of permeability was then determined as described in Materials and Methods. As shown in Fig. 5A, PGE_1_ increased the permeability of vascular barrier attained by HUVECs. The EP1 agonist ONO-DI-004 and the EP3 agonist ONO-AE-248 also decreased in transepithelial resistance as well as PGE_1_. The increase of the permeability was partially prevented by treatment with the HIF-1 inhibitor YC-1 or siRNA against HIF-1α (Fig. 7A). In addition, we also examined the permeability adopting transepithelial electrical resistance measurements. Transepithelial resistance was decreased 24 h after treatment with conditioned media derived from HUVECs treated with 1 µM PGE_1_, ONO-DI-004, or ONO-AE-248 treatment and the treatment with YC-1 or siRNA against HIF-1α prevented the increase of the permeability (Fig. 7B).

## Discussion

In this study, we demonstrated that the prostanoid PGE_1_ induced HIF-1 activation by increasing HIF-1α protein expression in an EP1- and EP3-receptor-dependent manner in the vascular derived cells HUVECs and HASMCs. In addition, we also indicated that PGE_1_ facilitated the initial step of angiogensis, stimulation and reorganization of basal lamina of endothelial cells.

Because effective-site concentrations of PGE_1_ in clinical settings are estimated to range from 100 to 5 µM, the concentration of PGE_1_ in the present study is close to clinically relevant levels (Cawello et al., 1995; Weiss et al., 2004). In this study, we used three types of PGE_1_; PGE_1_ and PGE_1_ conjugated to lipid microsphere and cyclodextrin, both of which are widely used in clinical field.

It is well known that the intracellular protein stability of both HIF-1α and HIF-2α is regulated by essentially the same HIF-α-prolyl hydroxylase-dependent mechanism (Hirota & Semenza, 2005; Schofield & Ratcliffe, 2005). PGE_1_ treatment did not induce the expression of the HIF-2α protein (Fig. 1D). The experimental results presented as Fig. 6A show that PGE_1_ treatment does not prolong the intracellular half-life of HIF-1α protein as long as prolyl hydroxylase DFX does. Together, the evidence suggests that PGE_1_ does not affect the protein stability of HIF-1α at least in either HUVECs or HASMCs. The other known mechanism of induction of HIF-1α protein expression is regulation of HIF-1α translation from mRNA (Dery, Michaud & Richard, 2005; Fukuda et al., 2002; Fukuda, Kelly & Semenza, 2003; Laughner et al., 2001). The neosynthesis of HIF-1α, especially induced by certain types of nitric oxide donors, growth factors, and agonists of GPCRs including acetylcholine, thrombin, and PGE_2_, is dependent on the activity of PI3K, AKT and mTOR (Fukuda et al., 2002; Fukuda, Kelly & Semenza, 2003; Gorlach et al., 2001; Hirota et al., 2004; Kasuno et al., 2004). Notably, it is reported that PGE_2_ induced HIF-1α protein expression by increasing HIF-1α protein translation from the mRNA in an EP1 receptor signal-dependent manner (Fukuda, Kelly & Semenza, 2003). As shown in Fig. 6C, PGE_1_-induced HIF-1α expression was inhibited by treatment with LY294002, PD98059, or GF109203X. Moreover, Fig. 6B indicates that PGE_1_ induces the neosynthesis of HIF-1α protein. In addition, PGE_1_ increased the expression level of HIF-1α mRNA but not that of HIF-1β mRNA in both HUVECs and HASMCs (Figs. 2A and 2B). Taking these observations together with our experimental findings that PGE_1_ does not prolong the half-life of HIF-1α protein (Fig. 6A) and that PGE1 enhances the neosynthesis of HIF-1α protein (Fig. 6B), we conclude that PGE_1_ activates HIF-1 mainly by increasing HIF-1α protein expression by enhancement of translation of HIF-1α in vascular-derived cells.

We showed that HASMCs express mRNA of all of the EP receptors at levels comparable to those of neuronal SH-SY5Y cells, which express all the EP receptors functionally (Ji et al., 2010) (Fig. 4A). We also demonstrated that HUVECs express mRNA of EP1, EP3, and EP4 prostanoid receptors (Fig. 4A). This result is consistent with previous findings (Amano et al., 2003; Kaneshiro et al., 2009). In both HASMCs and HUVECs, the EP1- or EP3-receptor-specific agonists ONO-DI-004 or ONO-AE-248 induced HIF-1α protein accumulation (Fig. 4B; lanes 2 and 4) and the HIF-1-downstream gene VEGF expression (Fig. 5A and 5B) and Glut1 expression (Figs. 5C and 5D) in both HASMCs and HUVECs. In contrast, the EP2- and EP4-receptor-specific agonists ONO-AE1-259-01 and ONO-AE1-329 failed to induce HIF-1α protein accumulation (Fig. 4B; lanes 3 and 5) and HIF-1 activation (Figs. 5A–5D). *K*i value of PGE_1_ for the other prostanoid receptor IP receptor is reported to be 33 nM, which is equivalent to that of EP1 receptor 36 nM (Narumiya, Sugimoto & Ushikubi, 1999). The evidence suggests that IP receptors may contribute at least partially to the activation of HIF-1 in HASMCs and HUVECs. However, we also demonstrated that the EP1- and EP3-receptor-specific antagonists ONO-8713 or ONO-AE3-240 significantly suppressed PGE_1_-induced HIF-1α expression (Fig. 4C, lanes 2 and 3) and induced VEGF mRNA expression (Fig. 5C). The evidence clearly shows that PGE_1_ induces HIF-1 activation in an EP1- and EP3-receptor-dependent manner at least in HASMCs and HUVECs. The results are consistent with the precedent studies (Fukuda, Kelly & Semenza, 2003; Ji et al., 2010), in which the critical role of the EP1 receptor in PGE2-induced HIF-1 activation was emphasized based on the evidence with an EP1 receptor antagonist in established cell lines. In addition to the EP1 receptor, we found that the EP3 receptor played a significant role in PGE1-induced HIF-1 activation from primary cultured cells of vascular origin. Signal transduction pathways of EP subtypes have been studied by examination of agonist- induced changes in second messengers such as cAMP, Ca^2+^, and inositol phosphates and of agonist-induced changes in activities of downstream kinases (Sugimoto & Narumiya, 2007). As shown in Fig. 6C, PGE_1_-induced HIF-1 activation is dependent on PI3K, MAPK, PKC, and mTOR in agreement with the observations that kinase inhibitors suppress the activation of HIF-1α protein expression and HIF-1 activation. This is also consistent with the precedent studies (Fukuda, Kelly & Semenza, 2003; Ji et al., 2010), suggesting that PGE_1_ utilizes the common mechanism of enhancement of HIF-1α translation.

Figures 7A and 7B illustrate that PGE_1_ increased the permeability of the vascular sheet reconstituted on collagen-coated inserts by HUVECs. The PGE_1_-HIF-1-VEGF axis may have contributed to this increased vascular permeability within a clinically relevant dose.

In a precedent study, it is reported that PGE1 induced VEGF and eNOS expression in an HIF-1-independent manner (Haider et al., 2005). It appears that there is a discrepancy between that report and our data but the reasons for the discrepancy are not currently clear. First, Haider et al. did investigate the expression of HIF-1α mRNA by conventional RT-PCR. We assayed the expression of HIF-1α mRNA by *q*RT-PCR but the change of the expression of HIF-1α mRNA was marginal (Figs. 2A and 2B). Second, it should be pointed out the basal expression of HIF-1α protein under normoxia was rather higher in their study compared to ours. Third, we confirmed the involvement of HIF-1 activation by inhibiting study adopting YC-1 and knocking down by siRNA againt HIF-1α (Figs. 3C and 7).

## Conclusions

We demonstrate that PGE_1_ induces HIF-1α protein expression and HIF-1 activation in a clinically relevant dose and formulation of drug and also provides evidence that the activity of multiple signal transduction pathways downstream of EP1 and EP3 receptors is required for HIF-1 activation by PGE_1_. Moreover, we indicate that PGE_1_ treatment induces the initial step of angiogenesis in HUVECs in an HIF-1-dependent manner.
